# How plausible is my model? Assessing model plausibility of structural equation models using Bayesian posterior probabilities (BPP)

**DOI:** 10.3758/s13428-025-02921-x

**Published:** 2026-02-23

**Authors:** Ivan Jacob Agaloos Pesigan, Shu Fai Cheung, Huiping Wu, Florbela Chang, Shing On Leung

**Affiliations:** 1 Edna Bennett Pierce Prevention Research Center, The Pennsylvania State University, University Park, PA USA; 2 Department of Psychology, Faculty of Social Sciences, University of Macau, Macao SAR, China; 3 College of Mathematics and Statistics, Fujian Normal University, Fuzhou, China; 4 Faculty of Education, University of Macau, Macau, China

**Keywords:** Model comparison, BIC, Posterior probability, Confidence set, Structural equation modeling

## Abstract

In structural equation modeling (SEM), one method to select the most plausible model from several candidates, or to compare one or more hypothesized models with similar alternatives on plausibility, is to compare the models using Bayesian posterior probability (BPP). BPP can be computed from the Bayesian information criterion (BIC) scores (Wu et al. *Multivariate Behavioral Research*, *55*(1), 1–16, 2020). This approach complements conventional goodness-of-fit indices such as the Comparative Fit Index (CFI), the root mean square error of approximation (RMSEA), and the standardized root mean square residual (SRMR) in giving concise BPP for assessing uncertainties among all models considered. It can also reveal evidence against a model otherwise hidden by these indices. However, Wu et al. *Multivariate Behavioral Research*, *55*(1), 1–16. (2020) did not provide guidelines on deciding the models that should be considered. To facilitate the use of BPP, we proposed a novel method for selecting this set of models, called *neighboring models*, to help researchers decide on the initial set. This novel method integrates seamlessly into the typical workflow for SEM analysis. Researchers can fit a model as usual and then use this method to assess whether it is the most plausible model compared with the neighboring models. We believe the proposed method will make it easier for researchers to make better-informed decisions when evaluating their models. We developed a user-friendly R package, modelbpp, to automate all the steps: generating the set of neighboring models, fitting them, and computing the BPPs, all in a single function.

In structural equation modeling (SEM), researchers grapple with the challenge of selecting the most suitable model from a set of candidates. This is typically done by computing various fit measures, such as the Comparative Fit Index (CFI, Bentler, 1990), the root mean square error of approximation (RMSEA, Browne & Cudeck, 1992), and standardized root mean square residual (SRMR), and then comparing models based on these measures or checking if they meet certain cutoff values. Although some fit measures, such as the CFI, do not involve null hypothesis testing, they still encourage making a discrete decision to select the “final” model. While this goal has practical value, the uncertainty in the decision is often ignored, similar to focusing solely on whether the estimate of a parameter is significant without considering the uncertainty reflected in the confidence interval of the estimate of this parameter. Moreover, some fit measures have the drawback that the saturated model always shows the “best fit,” making them unsuitable for assessing whether a hypothesized model has the “strongest support” from the data, unless it is the saturated model. Just as confidence intervals complement (or, some may argue, replace) null hypothesis significance testing in assessing a single parameter, we need similar methods to quantify the degree of uncertainty in model selection.

**Table 1:** Hypothetical BPP examples

							BPP With		BPP With
Model	$\chi ^2$	*df*	*p*	RMSEA	CFI	BIC	Unbiased prior	Prior	Prior
A	21.0	10	.021	.078	.971	78.12	.611	.20	.457
B	17.0	9	.049	.070	.979	79.32	.337	.40	.504
C	15.8	8	.045	.074	.980	83.31	.046	.20	.034
D	14.5	7	.043	.077	.981	87.20	.007	.20	.005

*Note*: In this hypothetical scenario, the number of observed variables is six, and the sample size is 180 (30 cases per variable). The baseline model is assumed to have a model $\chi ^2$ of 400, with $df = 15$. BICs are smaller than usual because they are computed from model $\chi ^2s$

Bayesian posterior probability (BPP), detailed in the next section, provides one such framework for model selection and evaluation by calculating the posterior probabilities of candidate models based on Bayesian information criteria (BIC) scores (Preacher & Merkle, 2012; Raftery, 1993; Wu et al., 2020). In the context of SEM, BIC is typically used to compare different models by penalizing the number of parameters in the model. Lower BIC scores indicate a better balance between model fit and complexity, while higher BIC scores indicate overfitting or worse model fit not justified by model parsimony. By extending BIC to form posterior distributions, researchers can evaluate the uncertainty associated with the models under consideration. BPP allows researchers to assign probabilities to different models, which range from 0 to 1, based on their BIC scores. If prior probabilities are equal, models with higher BIC scores will have lower posterior probabilities, indicating less support from the data. On the other hand, models with lower BIC scores will have higher posterior probabilities (assuming equal prior probabilities), suggesting stronger support from the data. By using BPP, researchers can make more informed decisions about model selection and evaluation by considering the uncertainty inherent in the modeling process. This approach provides a probabilistic framework (in a Bayesian sense) for comparing and selecting models, accounting for both model fit and complexity.

Despite the advantages of BPP, it is not yet popular in SEM applications, mainly due to difficulties in determining the set of models for comparison. The novel contributions of this manuscript include (a) proposing an automatic but adjustable workflow to evaluate a model by computing its BPP with respect to a reasonable selection of similar models, (b) presenting an R package, modelbpp, for researchers to adopt this workflow easily, and (c) proposing ways to adjust the workflow, such as the models to be compared to and the prior probabilities, if necessitated by theoretical reasons.

In this manuscript, we first give a brief introduction to BPP. We then present modelbpp, an R package for computing BPP for SEM models. We present the novel workflow for using the package that compares a hypothesized model with neighboring models that are 1 degree of freedom (*df*) *away*, that is, different from a hypothesized model by 1 *df*. We then proceed with an illustration of how to use the package using empirical examples. We extend the suggested workflow by presenting other features, including the ability to specify competing models, assessing neighboring models that are more than 1-*df* away from the hypothesized model, and visualizing the model relations and BPPs. We propose guidelines for reporting BPP results. Clear communication of findings is crucial, and we offer insights on how to achieve this. Like any fit measure, BPP has its limitations. We discuss these candidly to ensure informed usage. In closing, we emphasize the value of probabilistic model evaluation and encourage researchers to explore the power of modelbpp in their SEM endeavors.

## Bayesian posterior probability (BPP)

Suppose we have *m* candidate models under consideration, $M_1$, $M_2$, ... to $M_m$, of an arbitrary order. The prior probabilities, our subjective beliefs before accessing the data, of these *m* models are $\text {Prior}(M_1)$ to $\text {Prior}(M_m)$. Note that these are probabilities in the Bayesian sense, that is, they are measures of the degrees of belief for this set of *m* candidate models. They are not frequentist probabilities, so they are not about how “likely” each model is to be the true model “in the long run.” Let $\text {BIC}_i$ be the BIC of the $i^\text {th}$ models. As shown in Preacher and Merkle (2012) and Wu et al. (2020), the posterior probability of $M_i$, $\text {Posterior}(M_i)$, or the belief in $M_i$ after updating the prior belief based on the data, is given by:

1 $$\begin{aligned} \text {Posterior}(M_i) = \frac{e^{-\frac{1}{2}(\text {BIC}_i - \text {BIC}_1)}\text {Prior}(M_i)}{D}, \end{aligned}$$

2 $$\begin{aligned} D = \sum _{i = 1}^{m}e^{-\frac{1}{2}(\text {BIC}_i - \text {BIC}_1)}\text {Prior}(M_i). \end{aligned}$$

where $M_1$ is used as the reference model, to which other models are compared when computing the posterior probabilities. The model used as the reference model does not matter because $\text {Posterior}(M_i)$ will be the same after being normalized by *D*. If unbiased priors are used, then $\text {Prior}(M_i) = 1 / m$ and the computation simplifies to

3 $$\begin{aligned} \text {Posterior}(M_i) = \frac{e^{-\frac{1}{2}(\text {BIC}_i - \text {BIC}_1)}}{\sum _{i = 1}^{m}e^{-\frac{1}{2}(\text {BIC}_i - \text {BIC}_1)}} . \end{aligned}$$

We call the posterior probability computed from BIC the *Bayesian posterior probability* (BPP).

Table 1 illustrates the computation of BPPs for four models under consideration in a hypothetical scenario (SRMR not included because it cannot be determined solely by model $\chi ^2$ and model *df*). All four models have significant model $\chi ^2$, RMSEA less than .08, and CFI greater than .95. If we use RMSEA as the criterion, then we would select Model B, which has the smallest RMSEA. If we use CFI as the criterion, then we would select Model D, which has the largest CFI. However, they are all very similar numerically in RMSEA and CFI. If we use BIC as the criterion, then we would select Model A. This may sound odd because Model A has the highest RMSEA and lowest CFI, but we need to note that BIC takes into account model parsimony. As Wu et al. (2020) found, as sample size increases, the simplest true model (where parameters that are zero in the population are fixed to zero) tends to have the smallest BIC. This outcome aligns with the intended design of the BIC.

Although BIC and BPP result in the same rank order for the models with equal prior beliefs, the values of BIC cannot indicate *how* much the data favor Model A. Using BPP to assess the relative support from the data changes our conclusion from simply selecting one model. On one hand, Model A has the largest BPP (.611), making it the most plausible model among the four. However, Model B’s BPP is .337, meaning that while the data favor Model A, Model B cannot be completely ruled out, assuming equal prior beliefs. Instead of reporting only Model A (or B) and ignoring the others, a more informative approach is to tentatively focus on Model A while acknowledging that further empirical studies are needed to assess the plausibility of Model B relative to Model A.^1^

### Characteristics of BPP

BPPs have the following characteristics. First, the sum of the BPPs of the *m* models is equal to one, allowing them to be interpreted as probabilities. For example, the BIC score for Model A (78.12) is not meaningful on its own. However, the BPP (.611) can be interpreted as a probability in the Bayesian sense, representing the degree of belief in this model among the set of candidate models.

Second, a BPP is not an absolute measure for a model because its value depends on the models under consideration. A model can have a high BPP in one set but a low BPP in another. Instead, the BPP of a model represents its plausibility relative to the other models being considered (i.e., models $M_1$ to $M_m$). For example, if additional models are included in Table 1, Model A might no longer be the most plausible model. If Model A is ruled out for theoretical reasons and excluded from the set of candidate models (equivalent to setting its prior probability to zero), then Model B would become the most plausible model.

Third, if unbiased priors are used (i.e., all *m* models are equally plausible *before* knowing the data), the rank order of the models based on BPP is identical to the rank order based on BIC. Specifically, the model with the smallest BIC will have the largest BPP, as illustrated in Table 1.

### Advantages of BPP

BPP derived from BIC in SEM has the following advantages, some shared with BIC and some unique to BPP. The first advantage is model uncertainty assessment. BPP allows researchers to assess the uncertainty associated with model selection and evaluation by providing posterior probabilities for candidate models. This helps in understanding the relative support for each model based on the data on a meaningful metric, probability. Even though BPP rank order and BIC rank order can be identical, the difference in BIC between two models, such as the two models with the smallest BIC, is not directly interpretable. Researchers may select a model with the smaller BIC, without considering how *much* more plausible this model is compared to the other models under consideration. BPP encourages quantitative comparisons rather than, or in addition to, making discrete or dichotomous decisions. For example, two models may only differ slightly in BPP, say, .40 and .42. They are practically equally plausible, suggesting that we need more data to differentiate these two models instead of just claiming that the “final” model is the model with BPP .42.

The second advantage is the incorporation of prior probabilities. By incorporating prior probabilities, BPP enables researchers to incorporate existing knowledge or beliefs about the models under consideration. This helps make more informed decisions by combining prior information with a data-based model selection and evaluation process. BIC rank ordering assumes all models under consideration are equally plausible, which may not be reasonable in some situations. For example, let us assume that the column *Prior* in Table 1 represents our prior beliefs on the four models based on previous findings and theories. Model B is commonly agreed to be the most probable model and Model A is included just to see whether Model B should be further simplified. Therefore, we set the prior belief for Model B to .40, which is twice as plausible as each of the other three models. If these prior beliefs are taken into account, then Model B has the highest BPP (.504), though not so much larger than that of Model A (.457). Instead of resorting to a discrete decision to declare Model B the “final” model and ignore Model A, the findings suggest that more empirical studies are needed. Moreover, as shown later, we can assess the sensitivity of BPPs to prior probabilities to see how much the prior belief in a model affects the BPP.

The third advantage is the flexibility in model comparison. BPP provides a flexible framework for comparing models by assigning probabilities to each one. This allows researchers to consider multiple models simultaneously and evaluate their relative plausibility. In situations where two or more models have similar prior probabilities—such as when there are two or more dominant theories implying different, non-nested models that cannot be compared by a $\chi ^2$ difference test—BPPs can be used for comparison. The conclusion then is not simply which model is “true,” but rather the relative plausibility of the models.

The fourth advantage is addressing overfitting. BPP, like BIC, helps address overfitting by penalizing models with excessive complexity. By considering both model fit and complexity, BPP promotes evaluating model plausibility by balancing goodness of fit and model simplicity.

Last, BPP provides a full picture of the posterior distribution with respect to the candidate models. BPP generates a full posterior distribution of models, providing a comprehensive view of the relative probabilities of models under consideration. This allows researchers to explore the uncertainty in model selection and evaluation and make more informed decisions.

In sum, BPP offers a Bayesian probabilistic approach to model selection and evaluation in SEM, enhancing the transparency and robustness of the modeling process by considering both empirical evidence and prior knowledge.

### Complementing common fit measures with BPP

Given the advantages of BPP presented above, we propose to complement conventional fit measures such as CFI, RMSEA, and SRMR with BPP when evaluating a hypothesized model or comparing several probable models. We selected CFI, RMSEA, and SRMR, the three most popular measures of model fit for a discussion below and show how BPP can complement them in model evaluation.

#### Complementing CFI

CFI (Bentler, 1990) is computed as the proportionate reduction in (model $\chi ^2$ − model *df*) between the fitted model and the baseline model. It has the advantage of being bounded between 0 and 1, with a value of zero for the baseline model and one for the saturated model (and any models with model $\chi ^2$ less than model *df*). This bounded range makes CFI interpretable. However, it has two limitations. First, differences in CFI are not easy to interpret; for example, understanding what an increase or difference of .01 means can be challenging. Second, CFI generally increases when parameters are added (unless the decrease in model $\chi ^2$ is less than the increase in model *df*). Therefore, it cannot reliably determine whether a hypothesized model is better than a more complicated model. Evaluated by CFI alone, the “best” model is always the saturated model, making CFI unsuitable for model selection.

Like CFI, BPP also has a range of possible values (0 to 1). However, unlike CFI, this range represents probabilities, making the numerical values more interpretable. Additionally, the saturated model does not necessarily have the largest BPP because BPP accounts for model parsimony. As shown in Wu et al. (2020), even if the saturated model is included in the set of candidate models, the simplest true model will still be selected by BPP as the sample size increases. This is not the case for CFI, where the saturated model always has the largest CFI.

#### Complementing RMSEA

RMSEA (Browne & Cudeck, 1992) measures the error of approximation of a model. It has a lower bound of zero but no upper bound. Although some rules of thumb have been proposed—such as .08 or less being reasonable and .05 or less indicating a “close fit” (Browne & Cudeck, 1992, 239)—interpreting RMSEA values can be challenging, especially because of the lack of an upper bound. Additionally, like CFI, the saturated model always has the smallest possible RMSEA (zero), making RMSEA unsuitable for model selection.

BPP is closely related to RMSEA because both BIC, from which BPP is computed, and RMSEA are functions of the same three values: model $\chi ^2$, model *df*, and sample size. However, unlike RMSEA, which is an absolute measure, BPP quantifies relative support and is bounded between zero and one. As mentioned before, BPP takes into account model parsimony (measured by model *df*). Therefore, if a simpler model is the true model, BPP tends to support it as the sample size increases. In contrast, RMSEA, like CFI, always favors the saturated model because it always has the smallest possible RMSEA.

#### Complementing SRMR

SRMR (Bentler, 1995) summarizes the discrepancy between the observed sample statistics (covariances, as well as means if a model has a mean structure) and the implied statistics. However, similar to RMSEA, it has a lower bound of zero (perfect fit) and has no upper bound. The larger the value, the worse the fit is. Therefore, similar to RMSEA, the saturated model necessarily has the lowest possible SRMR (zero), and SRMR alone cannot be used for model selection. Unlike RMSEA, the computation of SRMR does not directly involve the model $\chi ^2$ and model *df*. Therefore, when used for model comparison, it cannot take into account model complexity, unlike RMSEA.

BPP can complement SRMR in the same way it complements RMSEA. It can quantify the degree of empirical support of a model relative to other models, and is bounded, unlike SRMR. Moreover, it takes into account model complexity, while SRMR cannot. If two models, one more complicated than the other, have the same value for SRMR, SRMR cannot be used to differentiate them, while BPP can. Last, just like the previous two measures, SRMR always favors the saturated model, even if the true model is a simpler model, while BPP will favor the simpler true model as the sample size increases.

#### Bayesian analogues of common fit measures

Although our focus is on model comparison via BPP, readers may also wish to interpret familiar indices such as RMSEA, CFI, and SRMR in a Bayesian context. Bayesian analogues of these indices have been proposed using the posterior predictive distribution (e.g., Asparouhov & Muthén, 2020). These approaches compute model misfit based on posterior predictive *p* values (PPP) and posterior summaries of discrepancy functions, yielding Bayesian versions of RMSEA, CFI, and SRMR. Conceptually, these indices assess the absolute fit of a single model to the data, analogous to their frequentist counterparts, but incorporating parameter uncertainty directly. In addition, Wu et al. (2014) introduced a novel relative–entropy–based posterior predictive checking approach that illustrates how alternative discrepancy functions can be employed in a Bayesian context to assess model fit in latent trait models. These approaches reinforce the general principle that any suitable statistic or function can be used to generate PPPs.

While these Bayesian fit indices offer a complementary perspective, they are not yet widely implemented in standard SEM software, unless one is fitting a fully Bayesian model, and their computation requires specialized procedures. In contrast, BPP provides a unified probabilistic measure for comparing a set of candidate models, subsuming considerations of fit and complexity. Nonetheless, we view Bayesian RMSEA, CFI, and SRMR as complementary tools for researchers interested in evaluating absolute fit in a Bayesian framework.

### Using CFI, RMSEA, SRMR, and BPP together

Despite the advantages of BPP, we do not believe it should replace conventional fit measures such as CFI, RMSEA, and SRMR. These measures complement each other and serve different purposes. If a model fits poorly according to CFI (e.g., a CFI of .50 or lower), RMSEA (e.g., an RMSEA greater than .50), or SRMR (e.g., an SRMR greater than .08), then even a model deemed highly plausible by BPP (.80 or .90) should not be selected. This is because BPP is computed with respect to a set of candidate models, meaning the most plausible model is only the most plausible among that set. It is possible that all these models fit poorly in the population. Therefore, BPP is most useful when we have one or more models that fit the data satisfactorily according to conventional fit measures, and we want to use BPP to measure the *strength* of empirical support for each candidate model.

In applied research, conventional fit measures such as CFI, RMSEA, and SRMR are typically used to justify the selection of the so-called “final” models. This is because CFI and RMSEA are computed without reference to other models (CFI is computed with respect to a baseline model, which is chosen for convenience and is rarely considered a plausible alternative). These measures are usually assessed against cutoff values (but see McNeish & Wolf, 2023, on a new approach to determine the cutoff values), ignoring the degree of uncertainty. This uncertainty can be quantified by *how much more plausible* a model is compared to similar models. BPP can serve this purpose because it is necessarily computed with respect to a set of candidate models.

In sum, conventional fit measures inform us about the degree of fit of a model, while BPP provides an assessment of the model’s relative plausibility compared to similar models. However, a practical question that may have hindered the use of BPP is how to determine the set of models to compare. Several procedures for this are presented in the next section. To facilitate the implementation of these procedures, we also illustrate how to use modelbpp, an R package developed for computing BPPs for models fitted by lavaan (Rosseel, 2012), a popular package for SEM analysis in R (R Core Team, 2024).

**Fig. 1:**
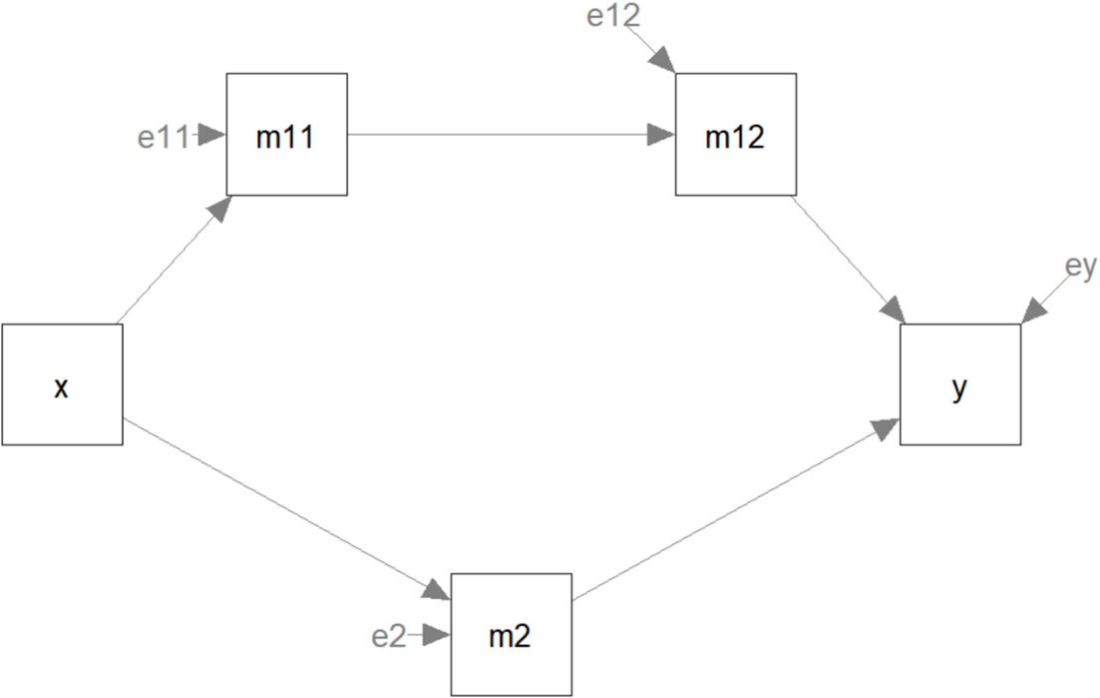
The path model in the hypothetical example

## A novel workflow for using BPP

Consider a typical scenario: A hypothesized model is fitted and needs to be evaluated for plausibility. Assuming this model has an “acceptable” fit according to criteria such as CFI, RMSEA, and SRMR, researchers would like to assess its plausibility within a set of similar models. One challenge in this approach is determining the set of models for comparison. In most typical applications of SEM, to convince readers that a model is worthy of discussion as a “final” model, researchers should demonstrate that (a) no fixed parameters need to be freed (e.g., no additional paths need to be added to the model), and (b) no free parameters should be fixed (e.g., no paths in the model should be removed). Therefore, we propose defining this set of similar models as those that differ from the fitted model by a one-*df* change. This idea can also be easily extended to include more models by considering models that differ from the hypothesized model by more than one *df*.

There are two subsets of these models. One is the set of models more parsimonious than the hypothesized model (i.e., having one more *df*), which we call the $+1df$ set. The other one is the set of models more complicated than the hypothesized model (i.e., having one less *df*), which we call the $-1df$ set. The set of models to be compared with the hypothesized models is the set of *neighboring models*. Therefore, to justify that a model is selected as the “final” model, this model should at least be more plausible than the $+1df$ set and/or the $-1df$ set of models.

If the main concern is to assess how much *more* plausible the hypothesized model is compared to more complicated models, and no paths are to be excluded because they are theoretically and/or empirically supported, it is reasonable to compare only against the set of $-1df$ models (e.g., models with one path or one covariance added). If it is also desirable to consider simpler models, to see how much more plausible it is to retain all the paths (or parameters in general) in the hypothesized model when compared to simpler models with one path removed, then the set of neighboring models should include both the $-1df$ and $+1df$ models (e.g., models with one path or one covariance removed, i.e., fixed to zero). We call these the 1*df*-*away* models. The set of all models, including the hypothesized model and the neighboring models, is referred to as the *model set* or the set of all candidate models under consideration.

This method to decide the models for comparison is easy to understand and easy to adapt. However, it is difficult to implement manually because researchers need to find and fit these models manually. One novel contribution of this manuscript is the development of the modelbpp package to make the identification of this set of models automatic, while the model set can still be adjusted by researchers if necessary. This is the suggested workflow for using the modelbpp package in this scenario:

1. Fit the hypothesized model using lavaan.
2. Call model_set() on the output of lavaan to do the following:
  Generate a list of models. If all paths are theoretically supported and should not be removed, then call model_set() to generate only the $+1df$ set. Otherwise, call it to generate the set of 1*df*-away models.
  Fit these models to the dataset.
3. Examine the BPPs of the original models and the neighboring models, either by printing the results or using a model network graph generated by model_graph().

This workflow is simple to use because only one more function call is needed. We first illustrate this workflow with a numerical example, and then examine how to use BPP in two real datasets. All data and script files used in the following sections can be downloaded from the OSF project of this manuscript. The package modelbpp is available on CRAN — a repository for R packages — and so can be installed just like most other R packages (details can be found on the CRAN page).

### A hypothetical numerical example

The dataset is in the Excel file data_path_model_se rial_parallel.xlsx. It has five variables: x (the predictor), m11, m12, and m2 (the mediators), and y (the outcome variable). The number of cases is 200. The following model is the hypothesized model, in lavaan model syntax (also shown in Fig. 1):
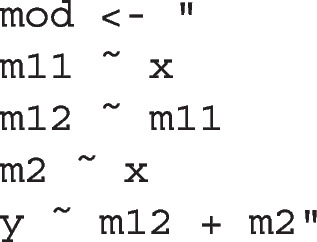

**Fig. 2:**
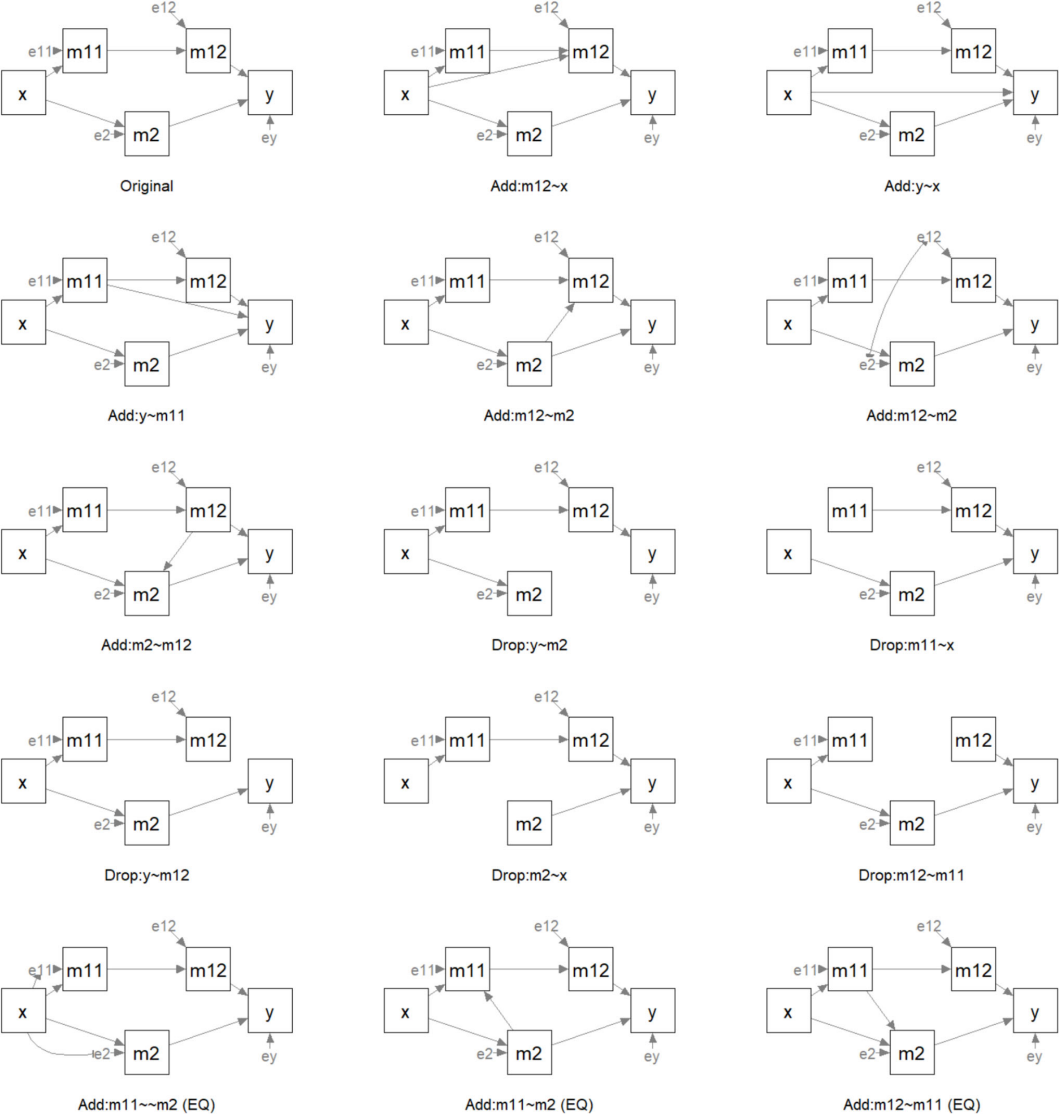
All models identified by model_set()

#### Fit the hypothesized model

We first fit this model in lavaan as usual and examine the output:
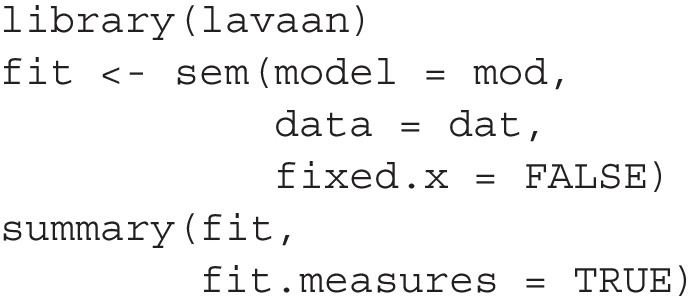


The model’s goodness-of-fit is satisfactory according to commonly adopted criteria: CFI = 0.976, RMSEA = 0.068, and SRMR = 0.056. Additionally, all path coefficients are significant, with *p* values less than 0.05.

In current practice, researchers might adopt this model as the final one. However, some models perform better on these fit measures (e.g., higher CFI, lower RMSEA, and/or lower SRMR). Examining these indices alone does not indicate how much more plausible this model is compared to other slightly different models.

#### Use model_set() to identify and fit neighboring models

The function model_set() can then be used on the output of lavaan:
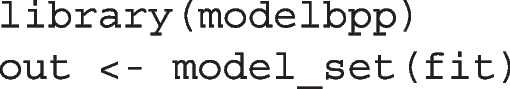


This function will identify all models different from the original model (fit in this example) by one *df*, such as those models with one path removed or added from the original model. By default, paths that will introduce a feedback loop will not be included (e.g., a path from y to m11, or to x). Moreover, a covariance between an error term of a variable and another variable that precedes it in a path (e.g., y and x), or the error term of this variable (e.g., m12), will also not be included. If theoretically meaningful, these variances and error covariances can be included by setting exclude_xy_cov to FALSE. If two or more such models are equivalent, determined by the procedure introduced by Bentler and Satorra (2010), only one of them will be arbitrarily selected and retained.^2^ The models fitted by model_set(), including the original model, are shown in Fig. 2. The three equivalent models, the lowest row, are marked by *(EQ)* in the figure.

After identifying these models, they will be fitted to the same dataset, BIC values extracted, BPPs computed, and then returned by model_set() for printing the results. By default, unbiased priors are used in computing BPP. That is, if the model set has 20 models, then the prior of each model, including the hypothesized model, is 1/20 or .05. We will show later how to set the prior probabilities directly.

#### Examine the BPPs

After running model_set(), we can print its results. This is an excerpt of the output:^3^
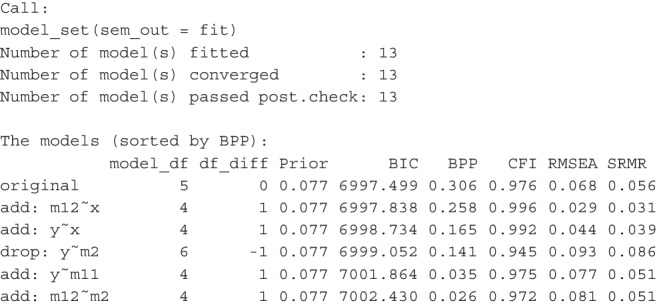


By default, the models are sorted according to BPPs, in descending order. The input model is labeled *original*. Other models are labeled by the changes compared to the original model. For example, the second model, |add: m12 x|, has the path from x to m12 added, and the fourth model, |drop: y m2|, has the path from m2 to y removed. The column df_diff is the difference between the original model and a model on model *df*. If the number is 1, then a model has one free parameter added, that is, it has one less model *df* than the original model. If the value is -1, then a model has one free parameter removed, that is, it has one more model *df* than the original model. The column Prior is the column of prior probabilities of models. With 13 models, the unbiased prior for each model is $1 / 13 \approx .077$. The column BIC has the BIC of each model, and the column BPP is the BPP for each model, computed from BICs and the prior probabilities. By default, three common fit measures, CFI, RMSEA, and SRMR, are also printed.

The results show that the hypothesized model indeed is the most plausible model, having a BPP of .306. However, it also shows that the support is not unambiguous. The BPP of the next model, with the path from x to m12 added, has a BPP value only slightly lower (.258). The BPPs of two other models, one has the path from x to y added, and the other has the path from m2 to y dropped, are .165 and .141, respectively.

**Fig. 3:**
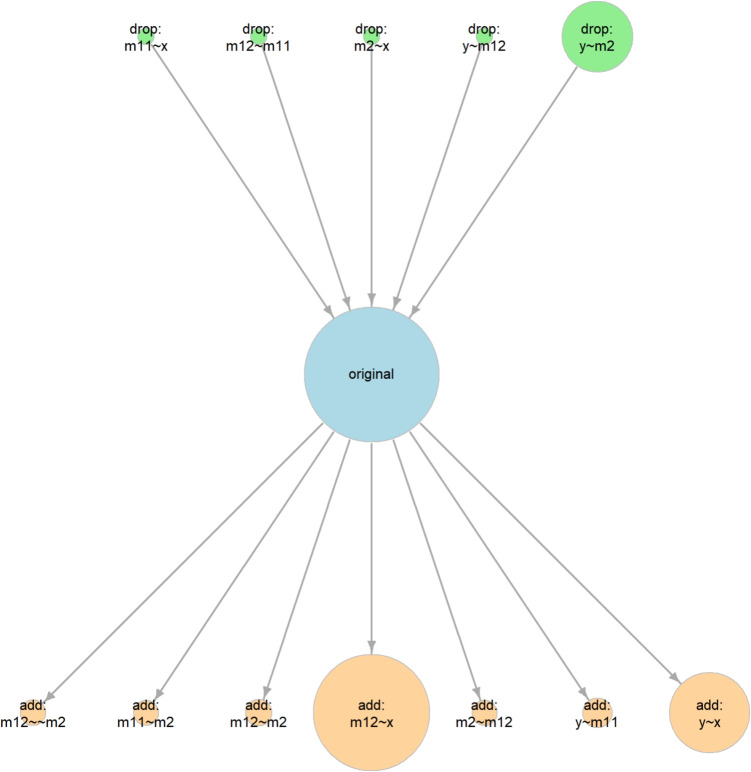
Visualizing the model relations and BPPs

The output also demonstrated how CFI and RMSEA could not be used to assess plausibility because of their general (though not exact) inverse relation with model *df*. SRMR could also not be used because it does not account for model parsimony. Among the top three models with the highest BPPs, the fitted model does not have the highest CFI, the smallest RMSEA, nor the smallest SRMR. Using CFI, RMSEA, and SRMR, it is difficult to justify why the fitted model is preferred. Even though one can argue that the fitted model is hypothesized on theoretical grounds, CFI, RMSEA, and SRMR cannot tell us *how much* more plausible the fitted model is. BPP, on the other hand, shows that, even not favoring the fitted model in prior belief, the data does support the fitted model more than the two other models.

The printout also shows that there is a cluster of three models that are equivalent to each other:
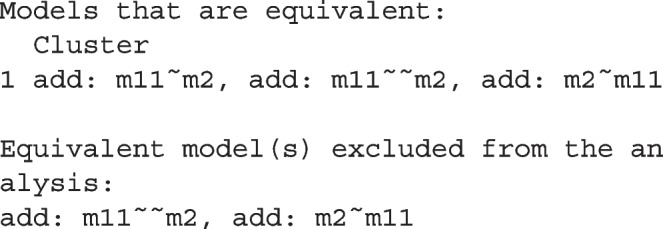


Because these models necessarily have identical values on all common fit measures, they should not be counted as three different models and so only one is retained. Which one to retain does not matter in the computation of BPPs because all three models have identical BIC and BPP values.

The function model_graph() can be used to visualize both the BPPs and the relations between the models:
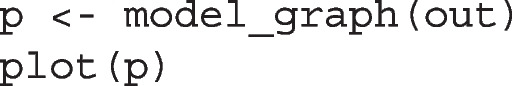


The output is a qgraph object (Epskamp et al., 2012), which can be plotted by calling the plot() function. The graph is shown in Fig. 3. By default, the width of a circle is scaled by the BPP of the model it represents (the larger the BPP, the larger the circle), to facilitate a crude comparison of BPPs.

### Set priors

One unique advantage of BPP is taking into account prior knowledge, if available. However, in practice, quantifying prior knowledge is not easy. Therefore, unbiased prior probabilities can be used, as in the previous example. If a model is the most plausible one *even if* unbiased priors are used, then it would still be the most plausible one if we assign a higher prior probability to it. We illustrate next how to use priors other than the unbiased priors.

#### Set the prior of the hypothesized model

An unbiased prior is usually not a *rational choice*, though it is convenient. A model is hypothesized for *some* reasons, typically theoretical. However, the prior belief in such a model is usually (a) strong enough that researchers believe it is worth studying, but (b) not so strong that empirical data is unnecessary. Therefore, we propose using $p_1$ as the prior probability of a hypothesized model, determined such that the prior of each remaining candidate model, $p_2$, is $0.80 p_1$. This means the prior of the hypothesized model is $(1 / 0.80)p_2$ or 1.25 times the prior of each other model in the set. The following formula can be used to find $p_1$:

4 $$\begin{aligned} p_1 = \frac{1}{.80m + .20} \end{aligned}$$

where *m* is the total number of models in the set.

In the present example, the total number of models is 13. Therefore, $p_1$ is approximately .094. We can set the prior of the original model by using the argument prior_sem_out, as shown below. If model_set() has been run, its output can be reused, without spending time to find the neighboring models again. We do this by setting model_set_out to the output of model_set():
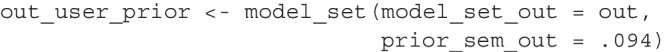


It will automatically set the priors for the remaining models.

This is an excerpt of the output, showing only the first six models:
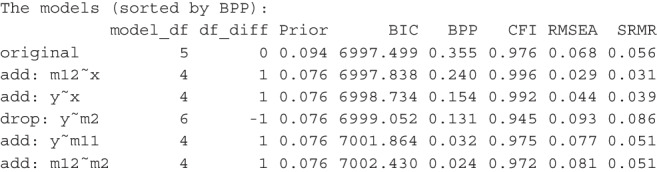


Naturally, the BPP of the original model is higher, increasing from 0.306 to 0.355. However, when compared to the next two or three models, the BPPs indicate that the hypothesized model is still not unambiguously supported by the data, even with an advantage in prior probability.

#### Set the target BPP and find the required prior

As a sensitivity analysis, we can also find the minimum prior required for the original model to have a desired BPP. That is, *how strong* our prior belief needs to be such that the model is as plausible as we want it to be. This minimum prior can be found by calling print() explicitly to print the output of model_set() and set bpp_target to the target BPP. This is an example with target BPP set to .80:
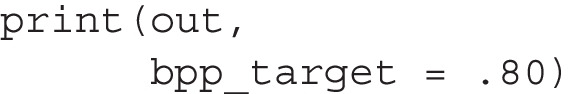


This is an excerpt of the output:
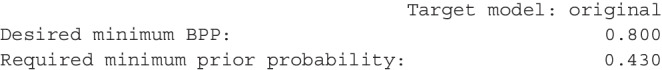


The target model is the name of the hypothesized model initially fitted, named *original* by default. It shows that the required minimum prior is .430, which is much larger than the unbiased prior. This suggests that, on the one hand, the data favor the original model. On the other hand, the support is not strong.

#### Interpreting the results

If we rely solely on conventional fit measures, we might settle for the original model, claiming it fits the data satisfactorily. While measures like CFI, RMSEA, and SRMR are “quantitative,” they compare the fitted models against either the baseline model (for CFI) or the saturated model (for RMSEA and SRMR). These measures tell us little about the degree of uncertainty and do not address how plausible this model is compared to other similar models, given the data.

The BPP analyses conducted above, using unbiased prior, theory-based prior, and target prior, allow us to interpret empirical support as a matter of degree in plausibility, rather than a simple “fits” or “does not fit” decision. Although the data favor the hypothesized model, a few other models are also plausible. Future studies can benefit from these results by designing studies specifically to differentiate these models empirically. For example, future studies may target these two paths, |m12 x| and |y x|, to see whether they really need to be added. Future studies may also use more reliable measures of y and/or m2 to determine whether the model with |y m2| dropped becomes less plausible.

## Illustration using a real dataset and a path model

To illustrate how to use modelbpp in a real scenario, we extracted a subset of the sample from a published study (Lau et al., 2023) and tested a path model. This model predicts anxiety (Anxiety) and physical health (Physical) by social support (Social) and four of the Big Five personality traits: extraversion (EXT), agreeableness (AGR), conscientiousness (CON), and emotional stability (EMO, the reverse of neuroticism).

It is proposed that social support mediates the effect of these personality traits on one or both well-being measures, while emotional stability has a direct path to both aspects of well-being due to its strong effects on well-being in general. The dataset and the R code can be obtained from the OSF page for this manuscript.

We first fit the hypothesized model in lavaan:
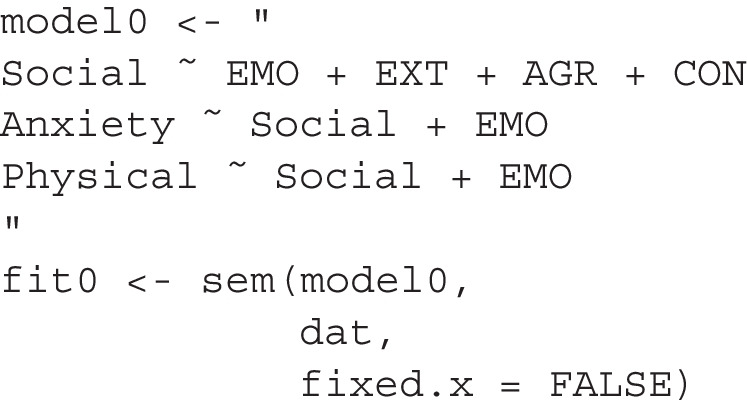


The model has a satisfactory fit according to common criteria (CFI = 0.974, RMSEA = 0.070, SRMR = 0.031), and all paths are significant. Typically, researchers might claim this as the final model supported by the data and proceed to interpret the results. However, as previously discussed, computing and comparing the BPPs of this model and its neighboring models can help assess the relative plausibility of this model compared to others. This procedure involves using the model_set() function as shown below:
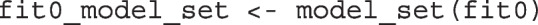


Due to the long names of the models and variables, short_names = TRUE is added to print the models with their short names:
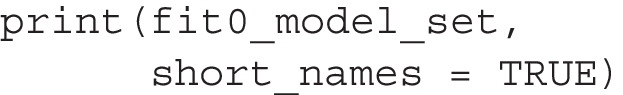


This is an excerpt from the printout
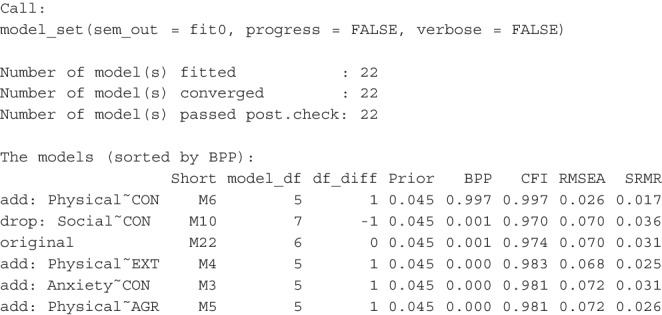


There are a total of 22 models, with 21 differing from the original model by one *df*.^4^ Using an unbiased prior, the original model (M22) is highly implausible (BPP = 0.001) despite its acceptable fit according to common measures. Model M6, with the direct path from conscientiousness to physical health added, is the most plausible (BPP = 0.997). Relying solely on fit measures may lead us to place too much confidence in the original model and overlook an important path that should be considered.

Although the model with the highest BPP also has the highest CFI, the smallest RMSEA, and the smallest SRMR, it is important to note that the second model (M10), with a BPP slightly higher than the original model (where the path from conscientiousness to social support is dropped), has a lower CFI and higher SRMR than the original model. Additionally, some models fit better than the original model in terms of CFI, RMSEA, and/or SRMR but have a lower BPP than the original model.

Therefore, model ranking based on BPP, while partly related to CFI, RMSEA, and SRMR, is not identical to rankings based on these fit measures. The numerical discrepancies in these fit measures cannot be directly translated into the assessment of relative model plausibility when using BPP, highlighting an advantage of BPP. Relying solely on model fit indices would have precluded the determination that the target model is not more plausible than certain neighboring alternative models differing by one *df*.

Suppose we believe there is a strong theoretical reason to adopt the proposed model, making unbiased priors irrational choices. In this case, we can set a target BPP for the hypothesized model and determine how high the prior probability needs to be for the model to achieve this target BPP. We can find this by adding bpp_target = .60 when printing the results:
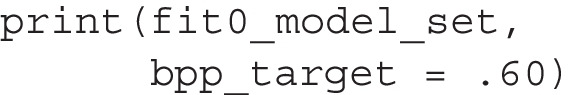


This is an excerpt of the printout:
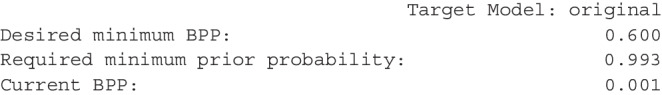


This shows that even if we only need a BPP of 0.60 for the hypothesized model, our prior belief needs to be extremely strong (0.993) to achieve this BPP, given the data. Thus, even strong theoretical reasons are difficult to justify the adoption of the original model in light of the data.

In this example, the natural step is to adopt the model with the path from conscientiousness to physical quality of life added and compute the BPPs again using the neighboring models of this modified model. Despite its acceptable goodness-of-fit by CFI, RMSEA, and SRMR, the hypothesized model is not plausible when compared to neighboring models.

## Illustration using a real dataset and a confirmatory factor analysis model

To illustrate how to use BPP in a more complex scenario with latent variables, we used the popular dataset from Holzinger and Swineford (cited in Kelley, 2007), available in the MBESS package (Kelley, 2007). We chose this version because it includes more variables for each of the three factors. The dataset contains scores from 301 students on tests of spatial ability, verbal ability, and (mental) speed ability, among other abilities. For illustration, we focus on these three abilities and use only the data from the Grant-White school, which includes 145 students. The subset can be generated with the following code:
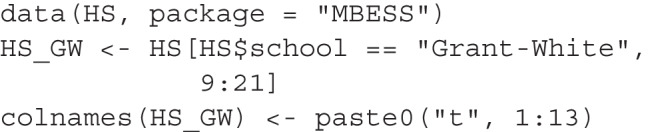


We first fit the hypothesized model, a three-factor confirmatory factor analysis (CFA) model:
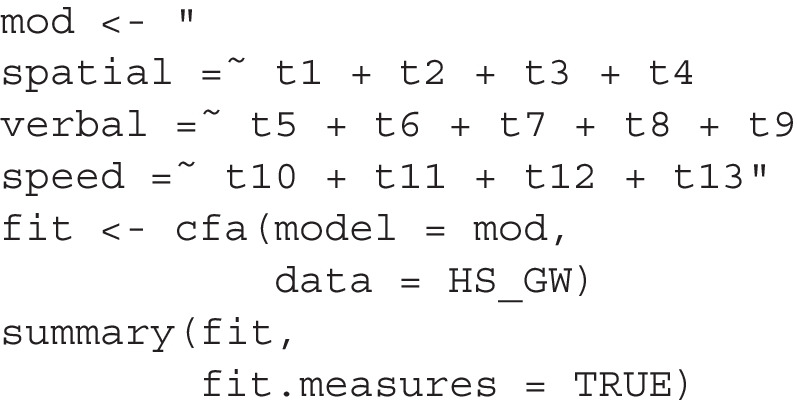


The fit is acceptable according to common criteria (CFI = 0.936, RMSEA = 0.075, SRMR = 0.065). All factor loadings are significant and have the expected positive sign.

For a CFA model, there are two main ways to add free parameters: by adding factor loadings or error covariances. While both types of changes can make the structure more difficult to interpret, adding error covariances is usually more problematic, except in longitudinal studies where each indicator has values from two or more time points. Additionally, the number of possible error covariances can be large (78 for a CFA model with 13 items). Therefore, by default, they are excluded when identifying neighboring models. If desired, they can be included by setting exclude_error_cov to FALSE.

This is the call to model_set():
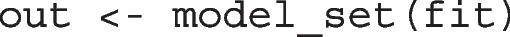


This is an excerpt of the printout:
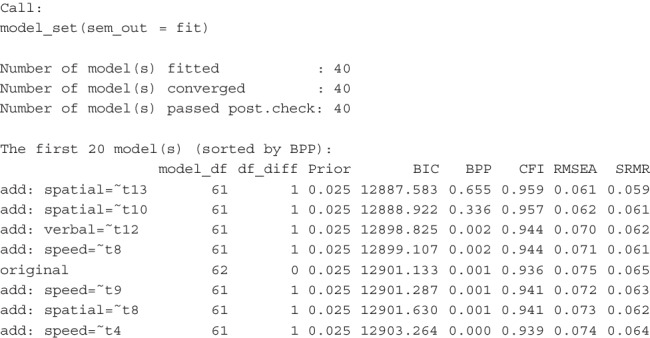


There are a total of 40 models, with 39 being neighboring models. Using an unbiased prior, the hypothesized CFA model with a simple structure is highly implausible (BPP = 0.001), despite its acceptable fit measures. Two models, both with a cross-loading added from spatial to an item in speed, are much more plausible.

Unlike previous examples, the rank order of the four models is more plausible than the fitted one remains the same even when using CFI, RMSEA, and SRMR. However, the numerical differences cannot be translated into plausibility based on BIC, illustrating the advantage of using BPPs. If we relied solely on fit measures, we would not realize that the hypothesized model is less plausible than some models differing by only one *df*. Consequently, we might adopt this model as the final one despite its relatively weak support from the data.

Suppose we want to argue that the simple CFA model is theoretically grounded and should therefore have a higher prior probability. In addition to using Eq. 4, we can perform a sensitivity analysis to find the minimum prior required to achieve a BPP of, say, 0.60 for the proposed CFA model:
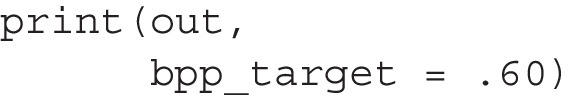


This is an excerpt of the printout:
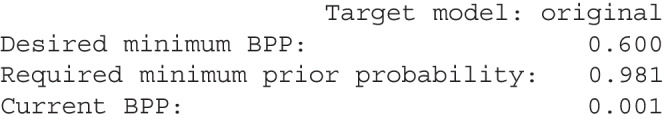


This shows that even if we only need a BPP of 0.60 for the hypothesized model, our prior belief needs to be unusually strong (0.981) to achieve this BPP, given the data. Thus, it is difficult to justify adopting the original simple CFA model given the data, even with a high prior probability.

How we should proceed in this case depends on the research context. We may drop t12 or t13 and rerun the analysis to see whether a simple structure is still plausible. Alternatively, we can interpret the model with cross-loadings included, suggesting that further studies revise these two items to make them more specific to the factor (speed) they are designed to measure. In all cases, BPP encourages us to confront the evidence against a hypothesized model, as this evidence may be hidden if we focus only on conventional fit measures.

## Other Features of modelbpp

### More than one hypothesized model

Researchers may not always have a strong preference for a single model. For example, in the path analysis model from the hypothetical numerical example (shown in Fig. 1), existing theories and findings might only suggest the two indirect paths and provide no guidance on the direct paths. Instead of favoring one model when setting the priors, it is more reasonable to set priors indicating that models with or without the direct paths from x to y, from x to m12, and from m11 to y are equally plausible. This can be done when calling model_set() by setting prior_sem_out to a named vector of numbers, with the names corresponding to the models as they appear in the printout of model_set(). Using the same rationale as above, we can use the following formula to determine the prior probabilities of the $k_1$ models, where $k_1$ is the number of models favored *a priori*:

5 $$\begin{aligned} p_1 = \frac{1}{.80k + .20k_1} \end{aligned}$$

With four such models, the prior is approximately 0.078 for each. We can then call model_set() and set the priors accordingly. Note that some labels need to be quoted because they contain spaces.
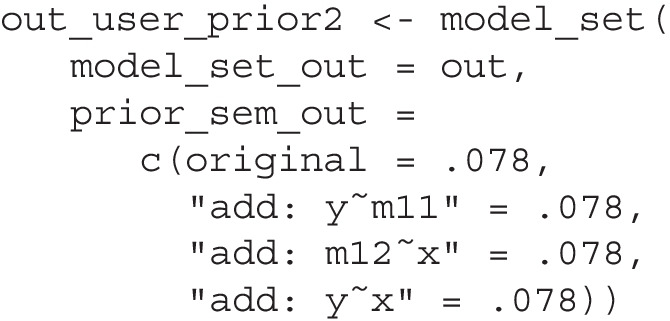


This is an excerpt of the output:
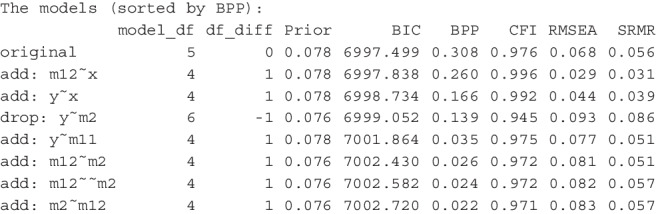


The hypothesized model remains the most plausible, although BPPs reveal that the support is not unambiguous. Researchers may still choose to adopt the hypothesized model as the final model. However, it is constructive to inform readers that further studies are needed to differentiate the top few models, especially since adding some paths (e.g., adding |"y x"|) may affect the parameter estimates of other paths in the model.

### Include models more than one *df* away from the original model^5^

**Fig. 4:**
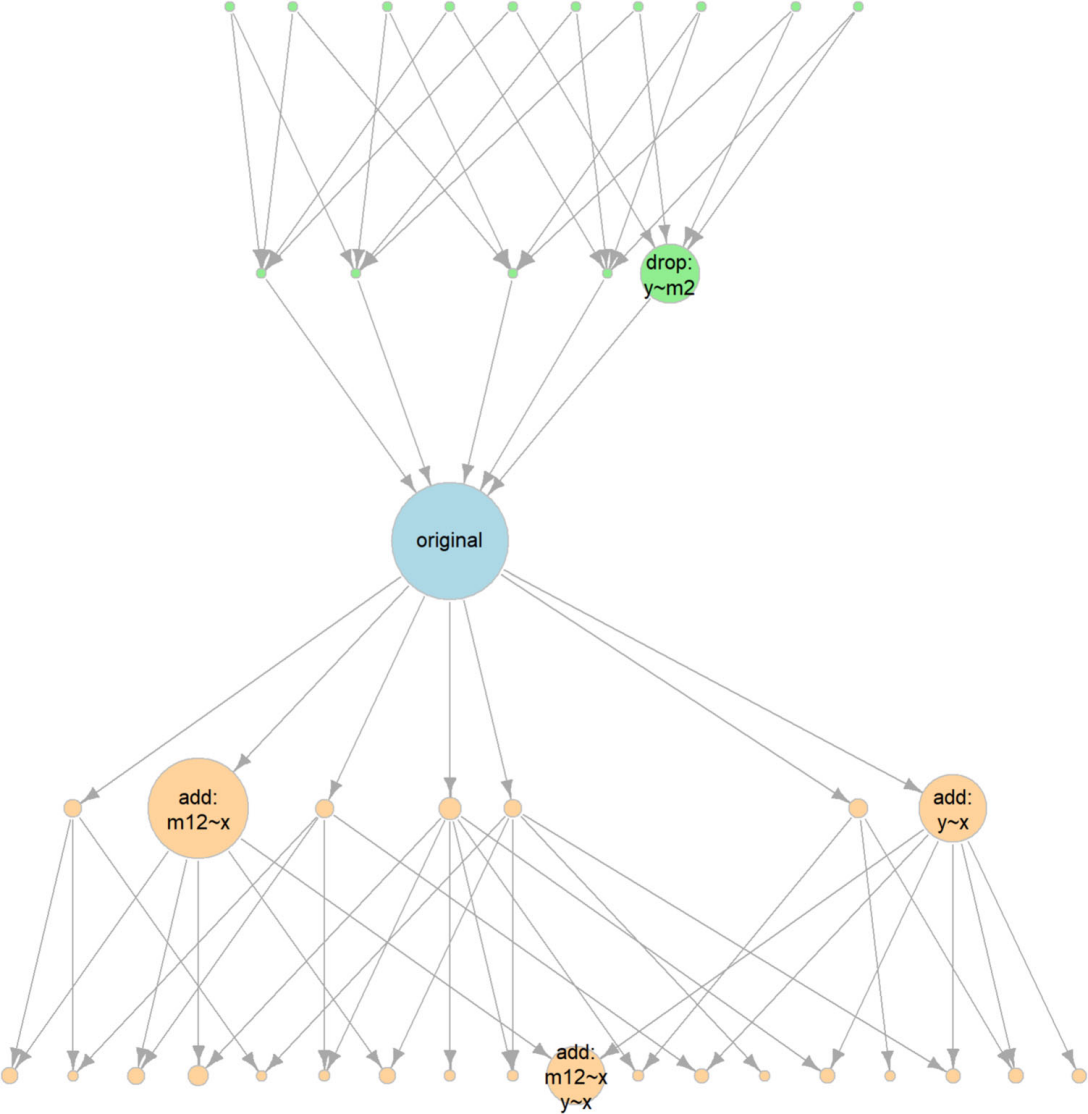
Network of models two or less degrees of freedom away

There may be situations where researchers want to assess the plausibility of a model by comparing it with models that differ by more than one *df*, such as models with two paths added or removed. This can be done by setting df_change_add and df_change_drop to the maximum desired changes in model $df$. The following call identifies all models that differ from the fitted model by one or two *df*:
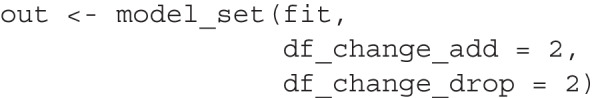


This is an excerpt of the output
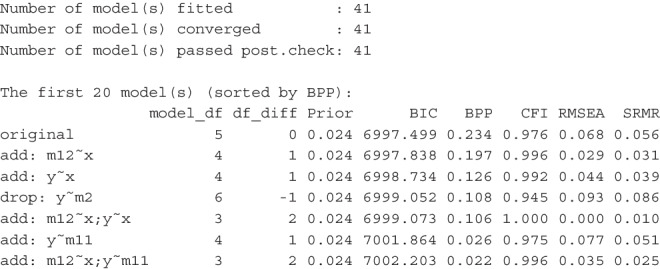


Note that the number of models increases exponentially as the number of *df* increases. The total number of models is 40 (excluding the fitted model). The fitted model still has the highest BPP. The 2-*df* away model with the largest BPP is the one with the paths from x to m12 and from x to y added, although the BPP is small (0.106).

Enlarging the range for neighboring models can be useful to assess whether a more plausible model may differ from the fitted model by more than one *df*. Using a larger set of neighboring models also increases confidence in the results by reducing the chance of missing a more plausible model.

The function model_graph() can also be used in this case.
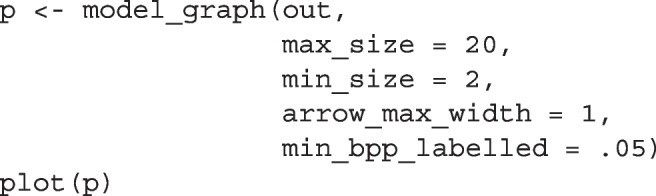


Due to the large number of models, it is advisable to customize the graph for better readability. There are many ways to customize it, and four arguments are used here. The arguments max_size and min_size control the sizes of the largest and smallest circles, respectively. Reducing max_size helps reduce or prevent overlapping between circles. Reducing min_size makes the circles of models with small BPPs smaller. Setting min_bpp_labeled to a small number (e.g., 0.05) hides the labels of models with BPPs less than this value, which is useful when we are only interested in models with BPPs larger than this threshold. Finally, arrow_max_width controls the width of the thickest arrows, which can be set to 1 or 0.5 when the number of arrows is large. The output is shown in Fig. 4.

#### Computing BPP without raw data

As long as the model can be fitted using summary statistics such as the sample covariance matrix, model_set() can be used without raw data. Therefore, BPP analysis can be performed on a model even if the raw data is not provided, as long as the covariance matrix being analyzed is reported. This allows users to analyze the plausibility of a model reported in previous studies, even if BPP analysis was not conducted at that time. The BPPs, compared to neighboring models, can be used in systematic reviews to provide a more comprehensive evaluation of empirical support for a model.

## Reporting the results

Using BPPs requires comparison with several, sometimes many, other models. When reporting the results, it can be inconvenient to include all the details, as is often the case with CFI, RMSEA, or SRMR. Therefore, we suggest reporting the following details for some common scenarios.

### One target model is evaluated

If only one target model is evaluated and compared with 1*df*-away models, researchers should report the following in the main text: (1) the BPP of the target model compared to the 1*df*-away models, and (2) the prior probability for the target model, or simply state that an unbiased prior is used. The code to reproduce the results of modelbpp can be included in the supplementary materials. If the support is not unambiguous, researchers can also report the BPPs of a few other models that should be investigated in further studies.

For example, this is how to report the results in the previous hypothetical example:

The fitted model has the highest BPP (.293) if compared to 14 1*df*-away models using an unbiased prior. However, the model with the direct path from x to m12 added also has a BPP of .247. We decided to tentatively adopt the fitted model as the final model.

In the Discussion section, the following can be added:

Although the fitted model is the most plausible among neighboring models, further studies are needed to examine the path from x to m12, using a larger sample and/or better measures of x and m12, to see whether this path should also be included.

#### Two or more target model evaluated

In this scenario, in addition to the information reported for the previous scenarios, the specification for all user-supplied target models need to be reported, as well as their prior probabilities.

For example, this is how to report the results in the previous example with more than one hypothesized model:

In addition to the hypothesized model, three models are also equally plausible theoretically: with the path from m11 to y added, with the path from x to m12 added, and with the path from x to y added. Therefore, their priors were set to $p_1$ or .078. The hypothesized model has the highest BPP (.309) if compared to 14 1 *df*-away models using an unbiased prior. However, the model with the direct path from x to m12 added also has a BPP of .261. We decided to tentatively adopt the hypothesized model as the final model.

In the Discussion, researchers can report again that further empirical studies are needed to differentiate these models.

## Compared to current practices

### Is it model modification in disguise?

Given that modification indices are used to identify less restrictive models and BPP is directly related to the model $\chi ^2$ difference between two models, one might argue that modification indices can serve the same purpose. Researchers could simply examine the reported modification indices. However, in our opinion, modification indices cannot achieve what BPP can for the following reasons.

First, there is no straightforward way to translate modification indices into meaningful units. Although models can be ordered, the degree of support from the data cannot be determined from the modification indices. For example, the hypothesized model always has a modification index of zero by definition, making it unclear how this can be used as an index for relative support from the data. Second, modification indices do not account for differences in prior beliefs about a model. Lastly, modification indices, which are closely related to the model $\chi ^2$ difference test, encourage binary decision-making. The plausibility of a model given the data and prior belief is a matter of degree, which BPP can adequately reflect.

### Can model $\chi ^{2}$ difference test achieve the same goal?

One might argue that instead of computing BPPs, we could perform a model $\chi ^2$ difference test between the hypothesized model and each of the neighboring models. If the hypothesized model fits significantly better than all the more restrictive models and not significantly worse than all the less restrictive models, then it is supported. While the model $\chi ^2$ difference test has its role in model comparison, especially when a difference or lack thereof is hypothesized *a priori*, this test is for making binary decisions. It does not indicate the degree of plausibility of a model compared to neighboring models. Moreover, if the comparison is not hypothesized *a priori*, conducting many tests risks capitalization on chance. Therefore, it cannot replace BPP for this purpose.

## Potential misuse and responsible application of modelbpp

Like other post-estimation tools, modelbpp can be misapplied if its exploratory components are treated as confirmatory evidence or used to justify *post hoc* theorizing. This concern parallels longstanding critiques of modification index-based model search, which can encourage capitalizing on chance and neglecting theory (e.g., MacCallum, 1986; Saris et al., 2009). Although modelbpp differs from modification indices by providing a probabilistically principled measure that incorporates model complexity via the marginal likelihood, the functional similarity – evaluating localized changes from a baseline – means that similar risks exist.

To promote responsible use, we recommend: (a) preregistering the candidate models when feasible; (b) reporting all models examined, not just those with high BPP; and (c) clearly distinguishing between exploratory and confirmatory analyses in reporting. We emphasize that the goal is not to fine-tune a model until fit indices (or BPP) are maximized, but to assess relative plausibility in a transparent and theory-informed manner.

One practical way to mitigate these risks is to embed modelbpp within a structured, theory-driven comparison process. Rather than relying solely on exploratory neighborhood searches, researchers can define in advance a limited, well-justified set of hypothesized models that represent competing theoretical perspectives. This safeguards against arbitrary model tinkering while still allowing the discovery of plausible nearby alternatives. Such an approach is formalized in what we call the *competing models framework*, described below.

### Competing models framework

An alternative to unrestricted neighborhood search is a competing models framework, in which the researcher specifies a set of theoretically defensible models in advance. Each model reflects a coherent theoretical account and may differ in structure or parameter constraints. The modelbpp workflow can then be applied to:

1. Estimate parameters for each hypothesized model,
2. Systematically generate and evaluate models that are one or more degrees of freedom ($\ge 1\,df$) away from each hypothesized model using the model_set function, and
3. Summarize the posterior probability distribution over all hypothesized models and their neighboring models using the model_set_combined function.

This approach balances theory-driven specification with openness to the possibility that nearby alternatives provide a better fit. For example, if two hypothesized models differ in theoretically important ways, evaluating their $1\,df$-away neighbors can reveal whether small, local modifications yield substantial gains in plausibility. Because BPP incorporates both fit and complexity, the framework naturally penalizes overfitting while quantifying the relative support for each model.

We view this theory-driven competing models approach as particularly valuable when strong prior expectations exist for two or more competing theoretically sound models, when the model space is large, or when the research question concerns the relative support for distinct theoretical positions rather than the incremental benefit of individual parameter changes.

## Limitations

### “True”, plausible, or approximating models?

As evident from the development of indices such as CFI and RMSEA, which are often used in place of the model $\chi ^2$ difference test in practice, it is commonly agreed that the models selected based on common fit measures or the models we hypothesize are not always the “true” model. The “true” model is defined as the simplest model that generates the data in the population. Even if the form of the model reflects the data-generating process (i.e., correct in the directions of the paths and all free parameters are indeed non-zero in the “true” model), it is still possible, and probable, that parameters fixed to zero in the selected model are not exactly zero in the population. BPP, and the BIC from which it is derived, can only assess the relative support for models given the data and prior beliefs. The most plausible model, even with a BPP of .90, is only the most plausible with respect to the models being compared. If all the models under consideration are too different from the “true” model, then BPP (and all other fit measures) cannot help us find the “true” model, regardless of the data.

Nevertheless, as sample size increases, BIC, and hence BPP, tend to select the *quasi-true* model from the set of candidate models (Burnham & Anderson, 2002). The *quasi-true* model is defined as the best approximating model, or models if there are two or more that equally approximate the true model.^6^ Therefore, as long as researchers understand that BPP measures the relative support of a model compared to other candidate models, BPP remains a useful measure of plausibility.

### The choice of candidate models

One difficulty in applying BPP as proposed by Wu et al. (2020) is the choice of candidate models. In their simulation study, it was possible to include nearly all models in the set due to the limited number of variables and the known data generation process. However, in practice, it is impossible to include all potential models due to the wide variety of possible model forms and the number of variables. To facilitate the application of BPP, we propose using 1*df*-away models as the default set of neighboring models and have developed a tool to identify them automatically. This is because, at a minimum, these models should be demonstrated to be less plausible than the tentative final model. We hope this approach will help researchers use BPP to complement existing measures. Nevertheless, to make the best use of BPP, all theoretically plausible models should be included in the model set.

### Favors models with weak paths omitted when sample size is small

By design, BIC, and hence BPP, takes into account model parsimony. As found in Wu et al. (2020) and Finch (2022), when the sample size is small and one or more non-zero parameters are weak (small in magnitude), the data generation model (the true model) may have a smaller BPP than the models with these parameters omitted (fixed to zero). Therefore, when using BPP for a model fitted to a small sample, this behavior should be considered. Thus, caution should be exercised when interpreting results in SEM with small sample sizes. Nevertheless, we emphasize that in both simulation studies, the true model tends to have the largest BPP as the sample size increases, which is a desirable behavior.

Although small sample sizes remain a challenge for all statistical methods, prior work has demonstrated that BPP can still perform reasonably well under certain conditions. For example, simulation studies by Wu et al. (2020) indicated that BPP tends to identify well-fitting or quasi-true models when sample sizes are moderate to large, and even when the sample size is relatively small (e.g., $N = 100$), provided that the magnitudes of the parameters (i.e., effect sizes) are large. These findings align with earlier demonstrations of the desirable asymptotic properties of BPP (e.g., Wagenmakers, 2007). Nevertheless, the degree to which these results generalize is conditional on the specific design features of the simulation scenarios (e.g., types of models, parameter magnitudes, sample sizes). Addressing this issue through a dedicated simulation study tailored to the neighborhood search workflow is an important direction for future research, but it is beyond the scope of this paper.

### Performance without known population model

When applying modelbpp to empirical data, the true population model is unknown. As a result, it is not possible to directly quantify the method’s accuracy in distinguishing correctly specified from misspecified models in these settings. Previous simulation studies in latent variable modeling contexts (e.g., Finch, 2022; Levy & Choi, 2013; Wagenmakers, 2007) have shown that BPP tends to identify well-fitting or quasi-true models when sample sizes are moderate to large, supporting its theoretical properties. However, these findings are necessarily conditional on the design choices of those simulations (e.g., model size, parameter magnitudes, sample sizes).

The present paper does not include new simulation work. Instead, our empirical examples are intended to demonstrate how the workflow can be implemented and interpreted in practice. We acknowledge that the generalizability of the results from these examples cannot be assumed without additional evidence. Future research could extend prior simulation work by explicitly designing scenarios that mirror the “neighborhood search” structure of modelbpp, including varying the number and nature of neighboring models, the degree of misspecification, and the sample size. Such studies would provide a more direct assessment of the workflow’s operating characteristics under conditions likely to be encountered in applied research.

### Distributional assumption

Currently, the derivation of BIC as an approximation of the Bayes factor relies on maximum likelihood estimation. Therefore, the performance of model evaluation based on BPP is unclear when other estimation methods are used, such as MLR and MLM (Savalei & Rosseel, 2022), or DWLS for ordinal observed indicators (Finney & DiStefano, 2013). Since BPP is not intended to estimate any objective frequentist probability but rather to evaluate the empirical support for a model within a set of candidate models, we believe BPP can still be used with other estimation methods, provided that strict cutoff values are not applied to BPP. Nevertheless, future studies are needed to assess the performance of BPP in model selection with nonnormal data.

### Sample size consideration

The performance of BPP – and model comparison workflows more generally – under conditions of small sample size is an important empirical question. Although prior work (e.g., Wagenmakers, 2007) has demonstrated that the BPP has desirable properties in large samples (such as consistency in selecting the quasi-true model), its performance in small samples is more variable and context dependent. As such, the sensitivity of the proposed workflow to sampling variability deserves systematic investigation. However, addressing this concern through a dedicated simulation study is beyond the immediate scope of this paper. At this stage, we do not offer a hard rule of thumb regarding minimum sample size, as this would depend on the complexity of the model and the nature of the data. We caution against over-interpreting BPP results from very small samples and suggest that users perform robustness checks (e.g., via bootstrap sampling or cross-validation) when feasible.

## Conclusion

We presented a novel and simple workflow to do BPP analysis (Wu et al., 2020) to quantify the empirical support for a model without making a dichotomous decision on model fit. This workflow can be conducted using the easy-to-use R package, modelbpp. In simple scenarios, just one function call (model_set) is sufficient. The package also offers options for tailoring the analysis to specific requirements, such as using a larger pool of neighboring models or setting user-defined prior probabilities. We hope this approach can make the evaluation of model fit more constructive. Researchers can still adopt a final model for discussion, but can also present other slightly different models that warrant further investigation in future studies. The modelbpp package and the BPP method can complement other goodness-of-fit measures in structural equation modeling, promoting a more constructive, non-binary evaluation of structural models.

## Data Availability

The data files and materials for all illustrations are available on the OSF project for this manuscript: https://osf.io/nmk5d/.

## Footnotes

1 BIC has also been used to compute Bayes factor for model comparison (Kass & Raftery, 1995; Raftery, 1995), and Bayes factor has gained popularity in psychological sciences (Heck et al., 2022). However, it is usually limited to comparing two models, and the value is less interpretable than a probability.

2 The method by Bentler and Satorra (2010) cannot yet be applied to a model with exogenous variables treated as fixed. When a path model is fitted with fixed.x = FALSE, the default of lavaan, it will be noted in the printout that models cannot be checked for equivalence.

3 The number of decimal places can be controlled by users. See the help page of print.model_set() for the details.

4 A full list of the models generated can be found from the output file at OSF. See the previous section on model_set() on models excluded by default.

5 The modelbpp workflow is not restricted to 1-*df* neighbors. Users can define any set of candidate models–regardless of complexity–and obtain BPP estimates for all. This flexibility also aligns with the *Competing Models Framework* described later, where theoretically motivated baseline models may differ by multiple degrees of freedom. In such cases, modelbpp can be used to compare these more substantially different models directly, or to explore their respective neighborhoods to identify plausible alternatives.

6 The degree of approximation is defined in Kullback–Leibler distance between a model and the true model, which is beyond the scope of this manuscript. Interested readers are referred to Sect. 6.4.2 of Burnham and Anderson (2002).

## Declarations

### Code availability

The source code for all illustrations are available on the OSF project for this manuscript: https://osf.io/nmk5d/. The source code of the package is available to the public on GitHub: https://github.com/sfcheung/modelbpp.

## References

[CR1] Asparouhov, T., & Muthén, B. (2020). Advances in bayesian model fit evaluation for structural equation models. *Structural Equation Modeling: A Multidisciplinary Journal,**28*(1), 1–14. 10.1080/10705511.2020.1764360

[CR2] Bentler, P. M. (1990). Comparative fit indexes in structural models. *Psychological Bulletin,**107*(2), 238–246. 10.1037/0033-2909.107.2.238 2320703 10.1037/0033-2909.107.2.238

[CR3] Bentler, P. M. (1995). *EQS Structural equations program manual*. Temple City: Multivariate Software Inc.

[CR4] Bentler, P. M., & Satorra, A. (2010). Testing model nesting and equivalence. *Psychological Methods,**15*(2), 111–23. 10.1037/a0019625 20515234 10.1037/a0019625 PMC2929578

[CR5] Browne, M. W., & Cudeck, R. (1992). Alternative ways of assessing model fit. *Sociological Methods & Research,**21*(2), 230–258. 10.1177/0049124192021002005

[CR6] Burnham, K. P., & Anderson, D. R. (2002). Model selection and multimodel inference: A practical information-theoretic approach (2nd ed.). Springer-Verlag. 10.1007/b97636

[CR7] Epskamp, S., Cramer, A. O. J., Waldorp, L. J., Schmittmann, V. D., & Borsboom, D. (2012). qgraph: Network visualizations of relationships in psychometric data. *Journal of Statistical Software*, *48*(4). 10.18637/jss.v048.i04

[CR8] Finch, W. H. (2022). Using BIC and aBIC to develop Bayesian posterior probabilities for latent variable models. *Psychological Test and Assessment Modeling,**64*(1), 22–47.

[CR9] Finney, S. J., & DiStefano, C. (2013). Nonnormal and categorical data in structural equation modeling. In G. R. Hancock & R. O. Mueller (Eds.), Structural equation modeling: A second course (2nd ed., pp. 439–492). Information Age Publishing.

[CR10] Heck, D. W., Boehm, U., Böing-Messing, F., Bürkner, P.-C., Derks, K., Dienes, Z., Fu, Q., Gu, X., Karimova, D., Kiers, H. A. L., Klugkist, I., Kuiper, R. M., Lee, M. D., Leenders, R., Leplaa, H. J., Linde, M., Ly, A., Meijerink-Bosman, M., Moerbeek, M., ..., & Hoijtink, H. (2022). A review of applications of the Bayes factor in psychological research. *Psychological Methods*. 10.1037/met0000454 10.1037/met0000454 35298215

[CR11] Kass, R. E., & Raftery, A. E. (1995). Bayes factors. *Journal of the American Statistical Association,**90*(430), 773–795. 10.1080/01621459.1995.10476572

[CR12] Kelley, K. (2007). Methods for the behavioral, educational, and social sciences: An R package. *Behavior Research Methods,**39*(4), 979–984. 10.3758/BF03192993 10.3758/bf03192993 18183915

[CR13] Lau, E. Y. Y., Cheung, S.-H., Li, C., He, S.-Y., Choi, H. F. H., Cheung, S. F., & Hui, C. H. (2023). Beyond material resources: Sleep well and be hopeful for less worry and better wellbeing. *Applied Research in Quality of Life,**18*(5), 2541–2560. 10.1007/s11482-023-10197-6

[CR14] Levy, R., & Choi, J. (2013). Bayesian structural equation modeling [Print version record]. In G. R. Hancock & R. O. Mueller (Eds.), *Structural equation modeling: A second course* (2nd ed.). Information Age Publishing, Inc.

[CR15] MacCallum, R. (1986). Specification searches in covariance structure modeling. *Psychological Bulletin,**100*(1), 107–120. 10.1037/0033-2909.100.1.107

[CR16] McNeish, D., & Wolf, M. G. (2023). Dynamic fit index cutoffs for confirmatory factor analysis models. *Psychological Methods,**28*(1), 61–88. 10.1037/met0000425 34694832 10.1037/met0000425

[CR17] Preacher, K. J., & Merkle, E. C. (2012). The problem of model selection uncertainty in structural equation modeling. *Psychological Methods,**17*(1), 1–14. 10.1037/a0026804 22268762 10.1037/a0026804

[CR18] R Core Team. (2024). R: A language and environment for statistical computing. R Foundation for Statistical Computing. Vienna, Austria. https://www.R-project.org/

[CR19] Raftery, A. E. (1993). Bayesian model selection in structural equation models. In *Testing structural equation models* (pp. 163–180). SAGE Publications

[CR20] Raftery, A. E. (1995). Bayesian model selection in social research. *Sociological Methodology,**25*, 111–163. 10.2307/271063

[CR21] Rosseel, Y. (2012). lavaan: An R package for structural equation modeling. *Journal of Statistical Software*, 48(2). 10.18637/jss.v048.i02

[CR22] Saris, W. E., Satorra, A., & van der Veld, W. M. (2009). Testing structural equation models or detection of misspecifications? *Structural Equation Modeling: A Multidisciplinary Journal,**16*(4), 561–582. 10.1080/10705510903203433

[CR23] Savalei, V., & Rosseel, Y. (2022). Computational options for standard errors and test statistics with incomplete normal and nonnormal data in SEM. *Structural Equation Modeling: A Multidisciplinary Journal,**29*(2), 163–181. 10.1080/10705511.2021.1877548

[CR24] Wagenmakers, E.-J. (2007). A practical solution to the pervasive problems of p values. *Psychonomic Bulletin & Review,**14*(5), 779–804. 10.3758/bf03194105 10.3758/bf03194105 18087943

[CR25] Wu, H., Cheung, S. F., & Leung, S. O. (2020). Simple use of BIC to assess model selection uncertainty: An illustration using mediation and moderation models. *Multivariate Behavioral Research,**55*(1), 1–16. 10.1080/00273171.2019.1574546 30932709 10.1080/00273171.2019.1574546

[CR26] Wu, H., Yuen, K.-V., & Leung, S.-O. (2014). A novel relative entropy–posterior predictive model checking approach with limited information statistics for latent trait models in sparse 2k contingency tables. *Computational Statistics & Data Analysis,**79*, 261–276. 10.1016/j.csda.2014.06.004

